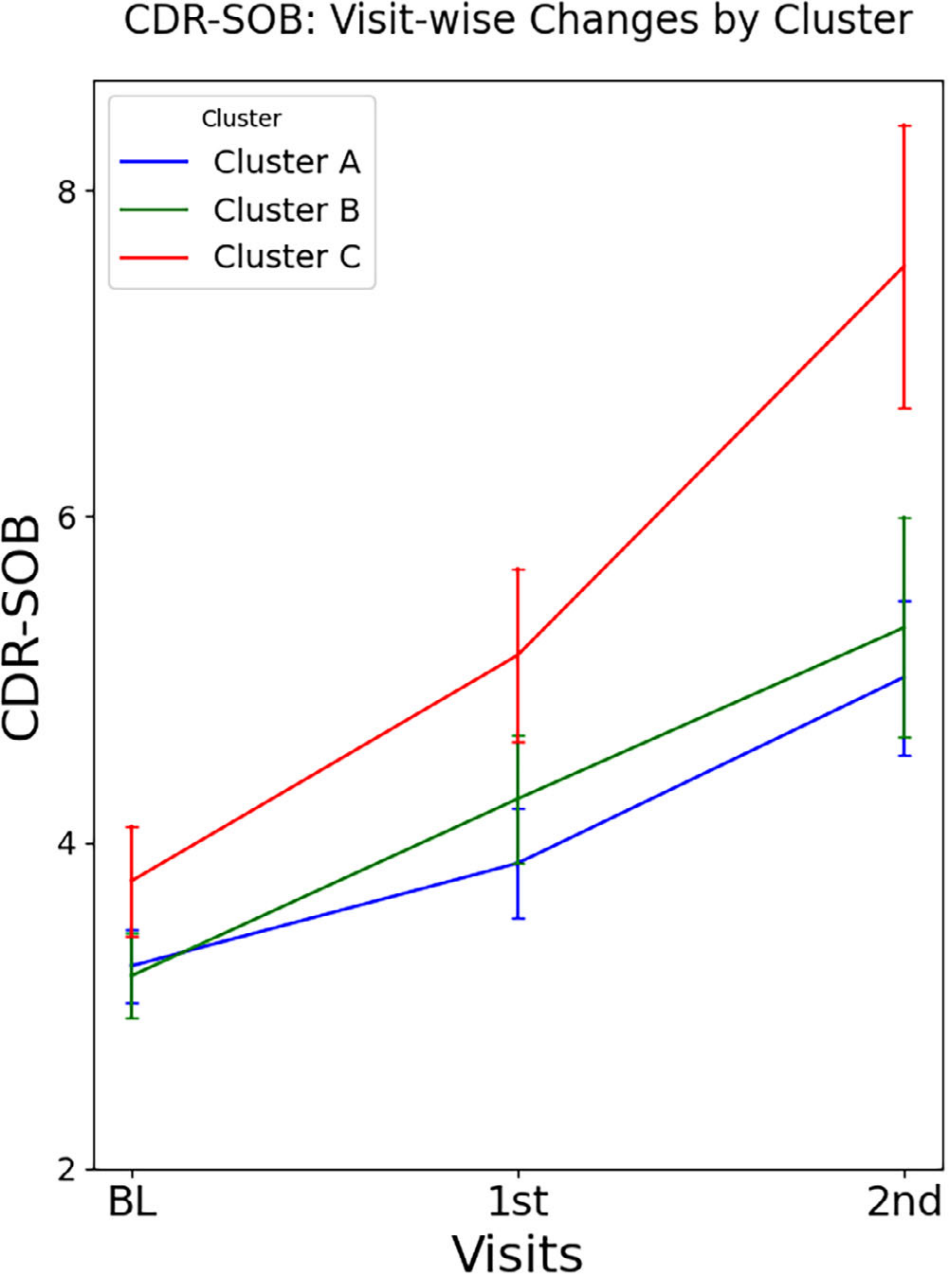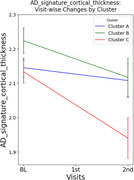# Distinct Longitudinal Trajectories of Alzheimer's Disease Subtypes Defined by Regional Amyloid Predominance Patterns

**DOI:** 10.1002/alz70856_104664

**Published:** 2026-01-07

**Authors:** Chung Hee Gwag, Min Soo Byun, Dahyun Yi, Hyejin Ahn, Yisak Kim, Yeongsoo Yoon, Gangwoo Kim, Yoonseok Oh, Bo Kyung Sohn, Seunghoon Lee, Minjae Kim, Jun‐Young Lee, Yu Kyeong Kim, Yun‐Sang Lee, Koung Mi Kang, Chul‐Ho Sohn, Kwangsoo Kim, Dong Young Lee

**Affiliations:** ^1^ Seoul National University Hoispital, Seoul, Korea, Republic of (South); ^2^ Seoul National University Hospital, Seoul, Korea, Republic of (South); ^3^ Seoul National University College of Medicine, Seoul, Korea, Republic of (South); ^4^ Seoul National University Medical Research Center, Seoul, Korea, Republic of (South); ^5^ Seoul National University, Seoul, Korea, Republic of (South); ^6^ Inje University Sanggye Paik Hospital, Seoul, Korea, Republic of (South); ^7^ Myongji hospital, Hanyang University College of Medicine, Goyang, Korea, Republic of (South); ^8^ Soonchunhyang University Seoul Hospital, Seoul, Korea, Republic of (South); ^9^ SMG‐SNU Boramae Medical Center, Seoul, Korea, Republic of (South)

## Abstract

**Background:**

It remains uncertain whether regional predominance patterns of amyloid accumulation are associated with neurodegeneration rates and the prognosis of Alzheimer's disease (AD) patients. This study aimed to examine differences in longitudinal neurodegeneration rates and clinical trajectories across AD subtypes defined through clustering analysis based on amyloid deposition pattern.

**Method:**

Participants were recruited from the Korean Brain Aging Study of Early Diagnosis and Prediction of Alzheimer's Disease (KBASE), started in 2014, Seoul, Republic of Korea. A total 149 amyloid‐positive cognitively impaired (CI) older adults consisting of mild cognitive impairment (*N* = 72) and mild AD dementia (*N* = 77) were included for this study. Participants underwent clinical and neuropsychological assessments at baseline, as well as at 1‐year and 2‐year follow‐ups. Multimodal neuroimaging, including [^11^C] PiB‐PET and MRI, was conducted at baseline and at the 2‐year follow‐up. Consensus clustering analysis was performed to identify AD subtypes based on regional predominance patterns of amyloid accumulation. Linear mixed‐effects models were used for longitudinal analyses.

**Result:**

We identified three AD subtypes with distinct regional predominance patterns of amyloid deposition among amyloid‐positive CI older adults using consensus clustering analysis: Cluster A (*N* = 62), characterized by orbitofrontal cortices and dorsal striatum predominance; Cluster B (*N* = 47), characterized by occipito‐temporal predominance; and Cluster C (*N* = 40), with fronto‐parietal predominance. At baseline, there were no significant differences in clinical diagnosis (i.e., MCI vs. AD dementia), clinical severity (i.e., CDR‐SOB), global cognition, or neurodegeneration biomarkers such as AD‐signature cortical thickness. However, in longitudinal analyses, Cluster C exhibited faster clinical decline and greater cortical atrophy in AD‐signature region compared to the other subtypes, even after adjusting potential confounders, including age, sex, education, apolipoprotein E4 positivity, and global amyloid burden.

**Conclusion:**

We found distinct longitudinal trajectories regarding both clinical progression and neurodegeneration rates among AD subtypes characterized by different patterns of amyloid predominance. Our findings suggests that an AD subtype with predominant frontal‐parietal amyloid deposition may have worse prognosis compared to other subtypes, underscoring the need for subtype‐specific therapeutic strategies. Further studies are required to elucidate the relationship between regional amyloid predominance patterns and progression rates of AD.